# Isolation, biochemical characterization, and primary structure and active site determination of *Dioscorea opposita* (‘Nagaimo’) oligopeptidase B

**DOI:** 10.1016/j.bbrep.2025.102071

**Published:** 2025-06-04

**Authors:** Sayaka Miyazaki-Katamura, Mami Chosei, Sota Tate, Tomohisa Sakaue, Takuya Yamane, Junko Suzuki, Shigeki Higashiyama, Iwao Ohkubo

**Affiliations:** a Department of Nutrition, School of Nursing and Nutrition, Tenshi College, Sapporo, 065-0013, Japan; b Department of Food Science and Human Wellness, College of Agriculture, Food and Environmental Sciences, Rakuno Gakuen University, Ebetsu, 069-0836, Japan; c Division of Cell Growth and Tumor Regulation Proteo-Science Center, Ehime University, Toon, 791-0295, Japan; d Department of Cardiovascular and Thoracic Surgery, Ehime University Graduate School of Medicine, Ehime, 791-0295, Japan; e Department of Biotechnology, Graduate School of Engineering, Osaka University, Suita, Osaka, 565-0871, Japan; f Department of Oncogenesis and Tumor Regulation, Osaka International Cancer Institute, Osaka, 541-8567, Japan; g Department of Pediatrics, Mikasa City Hospital, Mikasa, 068-2156, Japan

**Keywords:** Dioscorea opposita, Serine protease, Biochemical characterization, Primary structure, Mutagenesis

## Abstract

A protease was purified to homogeneity from *Dioscorea opposita “*Nagaimo” using ion exchange, hydrophobic and gel filtration columns, and its biochemical characterization including molecular weight, substrate specificity and kinetic parameters were determined. Protease activity was strongly inhibited by AEBSF, DCI and TLCK. The enzyme moderately inhibited by NEM and HgCl_2_. The enzyme activity inhibited by NEM and HgCl_2_ was restored with the addition of β-ME. These findings suggest that the enzyme is a trypsin-like serine protease, which is regulated by SH compounds. The N-terminal amino acid of this protease is blocked in an unknown manner. We determined the structure of the cDNA and deduced amino acid sequence of the protease from *D. opposita*. The cDNA was composed of 2420 nucleotides and encoded 751 amino acids in the coding region. The results indicated that this enzyme is an oligopeptidase B (OPB), consisting of a N-terminal region (M^1^ ∼ T4^7^), a N-terminal β-propeller domain (A^48^∼ L^465^), a connecting domain (K^466^ ∼ D^527^), a peptidase_S9 domain (P^528^ ∼ D^744^) and C-terminal region (R^745^ ∼ S^751^). The overall homology of amino acid sequences of *D. opposita* to *D. alata* and *D. rotundata* was 99.07 % and 97.07 %, respectively. The catalytically active amino acid sites [S^599^, D^684^, and H^719^] among these yam species were found to be highly conserved. Site-directed mutagenesis confirmed that these three the active center.

## Highlights

• A Protease was purified from *D. opposita* (Nagaimo) for the first time.
• The biochemical properties of the purified protease were clarified, and it was found to be a trypsin-type serine protease.
• Determination of the internal amino acid sequence of the protease revealed that this protease is Oligopeptidase B.
• The cDNA structure of this protease was first elucidated.
• The active center of this enzyme was identified using site-directed mutagenesis.

## Graphical abstract

**Figure:**
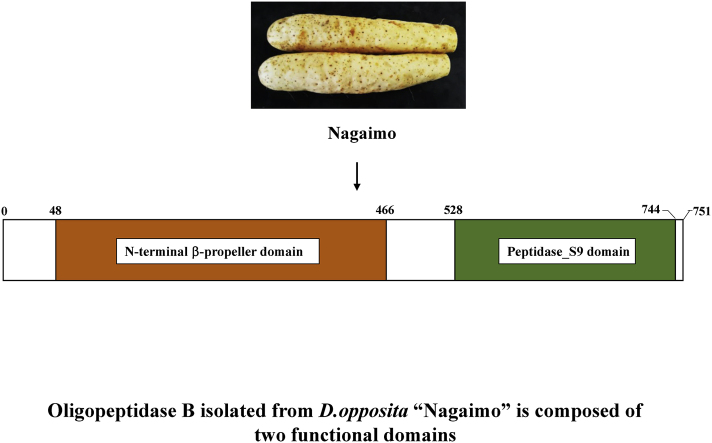

## 1 Introduction

The genus *Dioscorea*, which includes more than 650 species in the family *Dioscoreaceae*, is native to northeastern Asia and distributed worldwide [1]. *Dioscorea opposita* (Chinese yam, Nagaimo) is theorized to have been introduced into Japan by China during or before the 17th century, although there is no bibliographical confirmation.

Several varieties of Chinese yam are cultivated as economically important crops in Japan and are classified into three groups (nagaimo, tsukuneimo, and ichoimo) [2] based on their shapes. In particular, Nagaimo is eaten raw or cooked daily in Japan as a source of energy due to its richness in starch (13.9–27.1 %) [3,4], vitamins (B_1_, B_2_, biotin, and C) [4], and minerals (K^+^, P^5+^, Fe^2+^, and Zn^2+^) [4]. Nagaimo also contains dioscorin, which is composed of mannan-protein macromolecules that provide viscosity [5,6]. Dioscorin inhibits the inhibitory activities of carbonic anhydrase and trypsin [7].

Chinese yam (*D. opposita* “Nagaimo”) has been commonly used in Chinese medicine to strengthen stomach function, alleviate anorexia, and treat diarrhea [8]. Nagaimo tubers have recently been reported to exert several potential health benefits, including antihypertensive effects in spontaneously hypertensive rats [9], inhibitory activity against breast cancer cells [10], hypoglycemic effects [11], and anti-inflammatory effects [12].

Furthermore, *D. opposita* “Nagaimo” contains diosgenin, which is a steroid precursor (steroid sapogenin). Diosgenin has recently received considerable attention for its pharmacological and medical properties [[13], [14], [15]]. Several studies have reported the effects of diosgenin in the treatment of cancers [16,17], neurological diseases [18], hyperlipidemia [19,20], cardiovascular diseases [21], diabetes, obesity [22], and inflammation [23].

However, information on the biochemical properties of proteases originating from the tubers of *D. opposita* “Nagaimo” is scarce [24,25].

In this paper, we describe a procedure for the isolation of a trypsin-like serine protease from *D. opposita* “Nagaimo” tuber and report its biochemical and physiological properties as well as the cDNA structure and deduced amino acid sequence of oligopeptidase B (OPB). Furthermore, we identified the active site of this enzyme using site-directed mutagenesis.

## 2 Materials and methods

### 2.1 Plant and reagents

Chinese yams (*D. opposita* “Nagaimo”) harvested in the Hokkaido area (Hokkaido, Japan) were obtained from city markets and stored at −30 °C until use.

Fluorogenic peptide substrates, including benzoyl (Bz)-Arg-4-methyl-coumaryl-7-amide (MCA), Pro-Phe-Arg-MCA, benzyloxycarbonyl (Z)-Leu-Arg-MCA, Z-Gly-Pro-Arg-MCA, and Z-Arg-Arg-MCA, and protease inhibitors, including leupeptin, antipain, and *N*-[*N*-(L-3-*tran*s-carbxyran-2-carbonyl)-L-leucyl]agmatine (E−64), were purchased from the Peptide Institute (Osaka, Japan). The compounds 4-(2-aminoethyl)-benzenesulfonyl fluoride (AEBSF), tosyl-phenylalanyl-chloromethyl ketone (TLCK), and tosyl-lysyl-chloromethyl hydrochloride (TPCK) were obtained from Sigma-Aldrich Co. LLC (Tokyo, Japan).

The compound 3,4-dichloroisocoumarin (DCI) was obtained from Cayman Chemical Co. (Ann Arbor, MI, USA). Wide-View™ prestained protein size marker III (Mr 11–245 kDa), protamine, β-casein, and anti-His-tag antibody (Catalog no. 011–23091) were obtained from FUJIFILM Wako Pure Chemical Co (Osaka, Japan). ⟨α-Casein and γ-casein were obtained from MP Biochemicals LLC (Solon, OH, USA).

Bio-Safe Coomassie G-250, Quick start™ Bradford 1x dye solution, Mini-Protean TGX precast gel, UNOsphere Q, and t-Butyl-HIC were obtained from Bio-Rad Laboratories (Hercules, CA, USA). A gel filtration calibration kit, Phenyl-HP, and HiLoad Superdex 200 pg were obtained from GE Healthcare (Buckinghamshire, UK).

Sequencing-grade modified trypsin and RNA isolation kits, and Ex-Taq were obtained from Promega (Tokyo, Japan) and Takara Bio (Shiga, Japan), respectively. An anti His-tag antibody (Catalog no. 011–23091) was obtained from FUJIFILM Wako Pure Chemical Corporation. (Osaka, Japan).

All other chemicals were of analytical grade.

### 2.2 Assay of protease activity

Enzyme activity was determined by fluorometrically (Excitation at 360 nm; emission at 460 nm) by monitoring the liberation of AMC at 37 °C for 10 min in a reaction mixture containing 10 μl of 10 mM of Bz-Arg-MCA substrate, 100 μl of 0.5 M Tris-HCl buffer at a pH of 8.0, 10–20 μl of enzyme solution, and MiliQ water (18 mΩ) in a total volume of 1 m. The amount of AMC liberated was determined using a VersaFluor Fluorometer system (Bio-Rad, Hercules, CA, USA). One unit of activity was defined as the amount of enzyme that hydrolyzed 1 μmol of the substrate per minute.

### 2.3 Protein quantitation

Protein concentrations were determined by measuring the absorbance at 280 nm using a Shimadzu UV-1850 UV-VIS spectrophotometer (Kyoto, Japan). One mg of protein was defined as the concentration required to yield an absorbance of 1.0 (E ^0.1 %^_280 nm_ = 1.0).

### 2.4 Polyacrylamide gel electrophoresis (PAGE)

Samples were resolved by electrophoresis on 4–20 % polyacrylamide slab gels (Mini-Protean TGX) in 25 mM Tris, 192 mM glycine, and 0.1 % SDS at a pH 8.3, following the method of Laemmli [26]. Proteins in the gel were stained with Coomassie Brilliant Blue G-250.

### 2.5 Molecular weight determination

The molecular weight of the enzyme was determined using both sodium dodecyl-sulfate (SDS)-PAGE and gel filtration chromatography. As standard proteins for SDS-PAGE, Wide-View™ prestained protein size marker III (Mr 11–245 kDa) for SDS-PAGE was obtained from FUJIFILM Wako Pure Chem. Co. The gel filtration calibration kit (LMW) and immunoglobulin G were obtained from GE Healthcare, Bio-Sciences AB (Uppsala, Sweden) and Bio-Rad, respectively.

### 2.6 Purification of serine protease from D. opposita “nagaimo”

Step 1. Protease extraction

Nagaimo tubers (approximately 500 g) were peeled and chopped into small pieces, and immersed in one volume of cold 40 mM Tris-HCl buffer (pH 8.0) was added. The suspension then was homogenized using a food processor (Panasonic, Kusatsu, Japan).

The homogenized solution was centrifuged at 13,500×*g* for 30 min at 4 °C, and the supernatant obtained was dialyzed overnight against 20 mM Tris-HCl buffer (pH 8.0). The dialysate was recentrifuged at 13,500×*g* for 45 min at 4 °C to remove any precipitates.

Step 2. Ammonium sulfate fractionation

The supernatant was treated with solid ammonium sulfate at 35 % saturation. After gentle stirring for 30 min, the suspension was centrifuged at 13,500×*g* for 30 min at 4 °C, and the supernatant was brought to 65 % saturation by adding solid ammonium sulfate. After gentle stirring for an additional 30 min, the suspension was centrifuged at 13,500×*g* for 30 min at 4 °C. The pellet was suspended in a minimum volume of 20 mM Tris-HCl buffer (pH 8.0) and was dialyzed overnight at 4 °C against the same buffer.

Step 3. UNOsphere Q column chromatography

The supernatant was applied at a flow rate of 1.5 mL/min to a UNOsphere Q column (2.5 × 20 cm, bed volume: 100 mL) equilibrated with 20 mM Tris-HCI buffer (pH 8.0). After sample lording, the column was washed extensively with the equilibration buffer, and then a linear NaCl gradient combining 500 mL of the same buffer with 500 mL of 20 mM Tris-HCl buffer (pH 8.0) containing 0.4 M NaCl was applied. Fractions exhibiting a protease activity for Bz-Arg-MCA were collected, pooled, and dialyzed overnight at 4 °C against 20 mM Tris-HCl buffer (pH 8.0) containing 2.3 M ammonium sulfate.

Step 4. *t*-Butyl HIC column chromatography

The solution was applied at a flow rate of 1 mL/min to a *t*-Butyl HIC column (1.5 × 11 cm, bed volume: 20 mL) equilibrated with 20 mM Tris-HCl buffer (pH 8.0), containing 2.3 M ammonium sulfate. Non-adsorbed proteins were removed by washing with the same buffer, and a decreasing salt gradient combining 120 mL of the above-mentioned buffer and 120 mL of the above buffer without ammonium sulfate was applied. Fractions exhibited protease activity against Bz-Arg-MCA were collected and dialyzed overnight at 4 °C against 20 mM Tris-HCl buffer (pH 8.0) containing 0.75 M ammonium sulfate.

Step 5. Phenyl-HP column chromatography

**Fig. 1:**
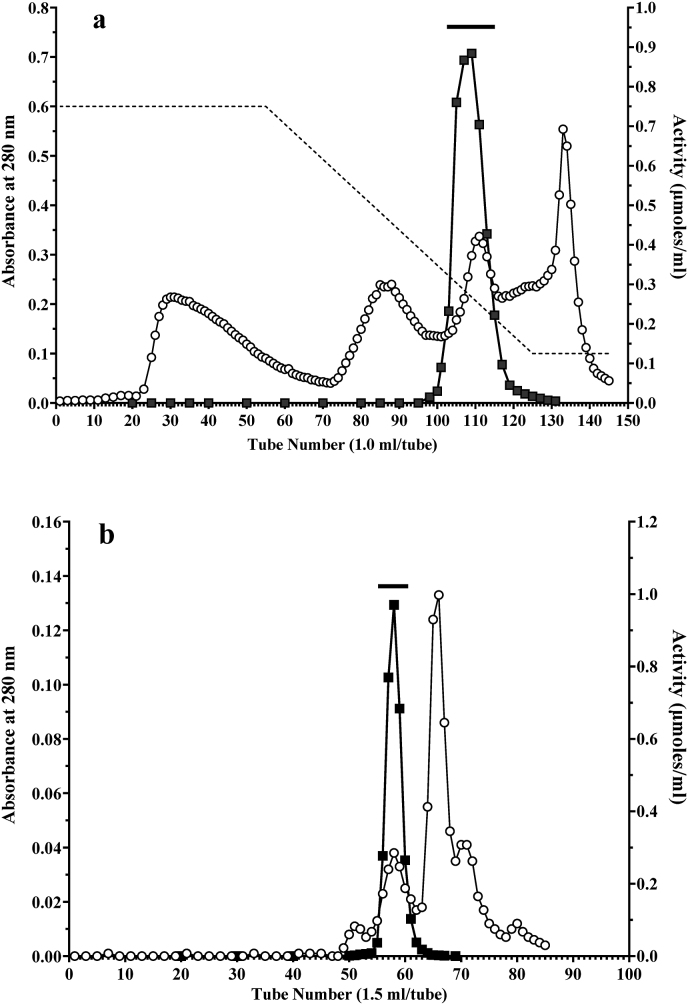
(a) Purification of a protease from *Dioscorea oppsita* “Nagaimo” using phenyl-HP column chromatography on a BioLogic DuoFlow system. A solution containing of 23.67 mg of protein was applied at a flow rate of 0.5 mL/min to a Phenyl-HP column (bed volume: 5 mL) equilibrated with 20 mM Tris-HCl buffer (pH 8.0) containing 0.75 M ammonium sulfate. Non-adsorbed proteins were removed by washing the same buffer, and a decreasing salt gradient was applied by combining 30 mL of the equilibration buffer and 30 mL of the same buffer without ammonium sulfate. Fractions with protease activity were collected and treated with solid ammonium sulfate at 75 % saturation. The obtained precipitate was suspended in a minimum volume of 20 mM Tris-buffered saline (pH 8.0). The solution was dialyzed overnight at 4 °C against the same buffer. The bar indicates the fractions that were pooled. Protein concentration (A280) (○); protease activity (■) and sodium chloride gradient (--). **(b)** Purification of a protease from *Dioscorea.opposita* “Nagaimo” using HiLoad superdex 200 pg column chromatography on a BioLogic DuoFlow system. A solution containing of 3.53 mg of protein obtained from the Phenyl-HP column was applied at a flow rate of 0.75 mL/min to a HiLoad superdex 200 pg column (1.6 × 60 cm) equilibrated with 20 mM Tris-buffered saline (pH 8.0). The bar indicates the fractions that were pooled. Protein concentration (A280) (○), protease activity (■) and sodium chloride gradient (--).

The solution was applied at a flow rate of 0.5 mL/min to a Phenyl-HP column (bed volume: 5 mL) equilibrated with 20 mM Tris-HCl buffer (pH 8.0) containing 0.75 M ammonium sulfate. Non-adsorbed proteins were removed by washing with the same buffer, and a decreasing salt gradient combining 30 mL of the above-mentioned buffer and 30 mL of the above buffer without ammonium sulfate. Fractions exhibiting protease activity against Bz-Arg-MCA were collected (Fig. 1a) and treated with solid ammonium sulfate to 75 % saturation. After 30 min, the suspension was centrifuged at 13,500×*g* at 4 °C for 30 min. The protein pellet was resuspended in a minimum volume of 20 mM Tris-buffered saline (pH 8.0), and dialyzed overnight at 4 °C against the same buffer.

Step 6. HiLoad Superdex 200 pg column chromatography in a BioLogic DuoFlow system

The solution was applied at a flow rate of 0.75 mL/min to a HiLoad Superdex 200 pg column (1.6 × 60 cm) equilibrated with 20 mM Tris-buffered saline (pH 8.0) (Fig. 1b). The fractions with protease activity were collected and used in subsequent experiments.

### 2.7 Kinetic analysis

Double-reciprocal (Lineweaver-Burk) plots were used to determine *K*m *and V*max values.

The concentration of an inhibitor that provided 50 % inhibition (IC_50_) was determined through a series of assays with varying inhibitor concentrations and a fixed substrate concentration of 100 μM.

### 2.8 Proteolytic activity of OPB on α-, β- and γ-caseins

The proteolytic activity of the purified protease (OPB) on hydrolysis of bovine milk protein was investigated in 20 mM Tris-HCl buffer (pH 8.0) for 1–24 h at 23 °C. The enzyme was added to α-, β- and γ-caseins at an enzyme/substrate ratio (E/S) of 1:10 in a reaction volume of 150 μl and incubated at 23 °C for 1–24 h. At regular intervals, 15 μl aliquots were mixed with 5 μl of SDS-PAGE loading buffer and heated for 10 min at 95 °C. The protein profiles of all samples were analyzed using 4–20 % SDS-PAGE.

### 2.9 N-terminal amino acid sequencing and internal amino acid sequencing of oligopeptidase B (OPB)

Purified protease (approximately 15 μg and 0.2 nmol) was subjected to SDS-PAGE on 4–20 % polyacrylamide gels and transferred to a polyvinylidene difluoride (PVDF) membrane (Bio-Rad). The membranes were stained with 0.1 % Coomassie Brilliant Blue R-250 in 10 % methanol–7 % acetic acid. The protein band corresponding to the expected size was cut out, washed with 50 % methanol, and then sequenced by automated Edman degradation using a Shimadzu PPSQ-33A instrument (Shimadzu Corp. Kyoto, Japan).

Purified protease (approximately 15 μg) was subjected to SDS-PAGE on 4–20 % polyacrylamide gel to determine the internal amino acid sequences.

The protein band stained with 0.1 % Coomassie Brilliant Blue R-250 was cut out and subjected to in-gel digestion using sequencing-grade modified trypsin, followed by LC-MS/MS analysis performed by Shimadzu Techno-Research (Shimadzu Corp., Kyoto, Japan).

### 2.10 Determination of the nucleotide sequence of OPB

Total RNA was isolated using an RNA isolation kit (Takara Bio, Shiga, Japan). After library preparation, fragmented RNA was reverse-transcribed to cDNA. Adapters were ligated to both ends of the cDNA fragments. After amplifying the fragments using polymerase chain reaction (PCR), fragments with insert sizes between 200 and 400 bp were selected. The cDNA libraries were sequenced at Macrogen (Seoul, Korea) using an Illumina NovaSeq 6000 System. Trimmed reads were assembled using the Trinity software. For the assembled genes, the longest of the assembled contigs was filtered and clustered into non-redundant transcripts using CD-HIT-EST. These transcripts were defined as unigenes. The obtained unigenes were used for subsequent annotation and ORF prediction. Target sequences were extracted. Since the 5′ end sequence of the target gene could not be obtained, the sequence was amplified using PCR and then sequenced. Reverse transcription was performed in a mixture containing 500 ng of total RNA and specific primers Nagaimo-F: 5′-CTCCGCTCGATCACTGTCTC-3′ and Nagaimo-R: 5′- TGATGGAGGAGCACTGCTAGA -3′ under the following conditions: 96 °C for 2 min, 40 cycles of 96 °C for 30 s, 60 °C for 30 s, 72 °C for 2 min, and 72 °C for 2 min using an Ex Taq (Takara, Shiga, Japan) and PCR system (Bio-Rad, Hercules, CA, USA).

### 2.11 Mutagenesis and cell-free protein synthesis of OPB

Based on the amino acid sequences determined by cDNA cloning, gene synthesis of the full coding region of oligopeptidase B was outsourced to Eurofins Genomics, Inc. The entire region was amplified PrimeStar Max (Takara Bio Inc., Shiga, Japan) with gene-specific primers (forward primer: ATGCTCCGCTCGATCACTGT, 6x-histidine-tagged reverse primer: TTAGTGATGGTGATGGTGATGCATGGAATTCACCAGTGGAATCA), and the PCR products were inserted into the pEU vector. Site-directed mutagenesis (S599A, D684A, and H719A) targeting the catalytically active domains of oligopeptidase B was performed using a PCR-based method with primers listed in Table 1. After plasmid amplification in *E. coli* strain JM109, *in vitro* translation (total volume: 250 μL) and cell-free protein synthesis were conducted using WEPRO7240H (Cell-Free Science Co., Yokohama, Japan) according to the manufacturer's instructions. A 200 μL aliquot of the soluble fraction containing the synthesized C-terminal histidine-tagged oligopeptidase B was collected after centrifugation at 10,000 g for 10 min at 4 °C. The supernatant was incubated with Ni Sepharose High-Performance histidine-tagged protein purification resin (50 % slurry, GE Healthcare, Madison, WI, Catalog No. 17526801) in binding buffer (20 mM phosphate buffer, 300 mM sodium chloride, and 20 mM imidazole, pH 7.5). After washing with binding buffer, the His-tagged oligopeptidase B was eluted with 20 μL of 500 mM imidazole in the washing buffer. The isolation and molecular weight of the synthesized proteins were evaluated through SDS-PAGE staining with Bio-Safe Coomassie and Western blot analysis using an anti-His-tag antibody (Catalog no. 011–23091., FUJIFILM Wako Pure Chemical Corporation, Osaka, Japan). The purified protein was immediately used for protease activity measurements as described above.

**Table 1:** Sequences of the primer used for site-directed mutagenesis of OPB of *Dioscorea Opposita*‘Nagaimo’.

Primer name		Primer sequence
Ser^599^ → Ala	+	GGG-AGA-**GC**T-GCA-GGA-GGC-TTG-CTT-ATG-GGT
(AGT → GCT)	–	TCC-TGC-A**GC**-TCT–CCC–ATC-TAT-ACA-TAA-TTT
Asp^684^ → Ala	+	TTG-AAT-G**C**T-ACC-CGT-GTT-ATG-TAC-TCG-GAG
(GAT → GCT)	–	ACG-GGT-A**G**C-ATT-CAA–CCC–TGC-TGT-AAC-AAG
His^719^ → Ala	+	GCA-GGG-**GC**C-TCC-TCA-AAG-TCA-GGA-AGA-TTT
(CAC → GCC)	–	TGA-GGA-G**GC**-CCC-TGC-AGC-TAG-TTC-ACA-TTT

*Note.* The codon changed is underlined and mismatched basepares are shown in bold letters. The sense oligopeptides are indicatedes by + and the antisense oligonucleotides by -.

### 2.12 Statistical analysis

Statistical analyses were performed using the GraphPad Prism 8 software (GraphPad Software Inc., La Jolla, CA, USA). Results are presented as the mean values ± standard error (SEM) of triplicate experiments. Statistically significant differences were determined using Student's t-tests, and the P-values are presented as follows: ns, not significant; ∗∗∗*p* < 0.001; ∗∗∗∗*p* < 0.0001.

## 3 Results and discussion

### 3.1 Purification of protease

**Fig. 2:**
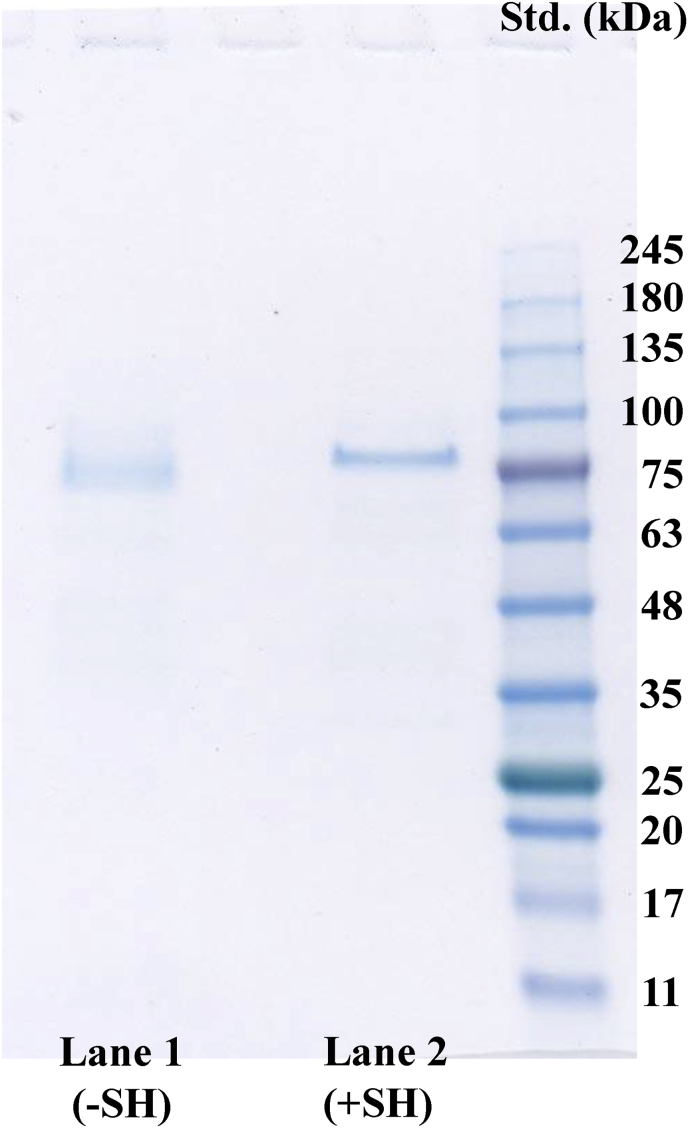
Sodium dodecyl sulfate-polyacrylamide gel electrophoresis (SDS-PAGE) of serine protease purified from *Dioscorea. opposita* ‘Nagaimo’. Electrophoresis of the purified protease was performed on a Mini Protean TGX precast gel (any size) in the presence of SDS and the gel was stained with Coomassie Brilliant Blue G-250. Lane 1 contained 2 μg of the purified enzyme in the absence of β-ME, and lane 2 contained the same amount of the protein in the presence of β-ME. Wide-View™ Prestained Protein Size Marker III protein standards (Fuijifilm Wako Pure Chemical Corporation) were used to estimate the molecular weights. The molecular weights of the maker proteins are indicated on the right-hand side.

The protease was purified from approximately 500 g of *D. opposita* “Nagaimo” using UNOsphere Q, t-Buthyl-HIC, Phenyl-HP, and HiLoad Superdex 200 gp chromatography (Fig. 1a and b). In a typical purification procedure (Table 2), the protease was purified approximately 2133-fold with an 8.9 % yield recovery. The final enzyme preparation migrated as a single band on SDS-PAGE in the absence and presence of β-ME (Fig. 2). The overall enzyme yield of the enzyme from 100 g of *D. opposita* “Nagaimo” was approximately 0.51 mg.

**Table 2:** Purification of protease from Nagaimo.

Step	Total absorbance	Total activity	Specific activity	Purification	Yield
	(280 nm)	(nmol)	(nmol/mg/min)	(-fold)	(%)
Supernatant	4206.0	32,975	7.84	1.00	100.0
(NH_4_)_2_SO_4_	1080.0	22,604	20.93	2.67	68.5
UNOsphere Q	101.0	14,867	147.19	18.77	45.1
*t*-Butyl HIC	23.67	11,130	420.22	53.60	33.8
Phenyl-HP	3.53	10,472	2966.45	378.37	31.8
Hilord 200	0.225	2950	16,720.90	2132.77	8.9

∗Protein concentration was measured by absorbance at 280 nm in 1-cm light path, and 1 mg of protein was defined as the concentration required to yield an absorbance of 1.0 (E ^0.1 %^_280 nm_ = 1.0).

### 3.2 Biochemical characterization of purified protease

#### 3.2.1 Molecular weight

The molecular weight of the purified protease from *D. opposita* “Nagaimo” was determined to be approximately 75,000 and 81,000 on SDS-PAGE in the absence and presence of β-ME, respectively (Fig. 2). The native molecular weight of the enzyme was determined using gel filtration and calculated by comparing with log Mr and *K*_av_ of standard proteins, bovine serum IgG (Mr 155,000), Conalbumin (Mr 75,000), Ovalbumin (Mr 44,000), carbonic anhydrase (Mr 29,000) and ribonuclease A (Mr 13,700). The purified enzyme had a molecular weight of approximately 75,000, indicating that it is as a monomer under native conditions.

Tsukamasa et al. [24] purified a protease from Nagaimo using casein as substrate, and reported the molecular weight of the purified protease from Nagaimo to be 370,000 using gel filtration. While in our experiment using a different substrate, Bz-Arg-MCA, the molecular weight of purified protease was calculated to be 75,000 in gel filtration. This result suggests that the protease purified by Tsukamasa et al. [24] and the protease we purified in this study are likely to be different proteases.

#### 3.2.2 Substrate specificity and kinetic parameters

**Fig. 3:**
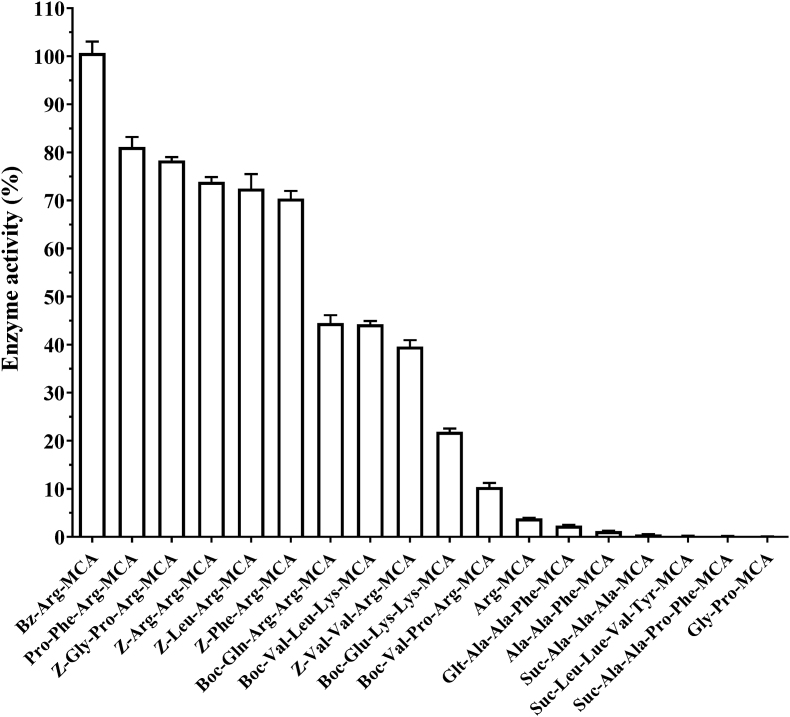
Substrate specificity of purified protease from *Dioscorea. opposita* “Nagaimo”. Enzyme activity was determined at 37 °C for 10 min in 50 mM Tris-HCl buffer (pH 8.0) with 0.15 μg of purified protease and 100 μM of substrate solution. Each value represents the mean ± standard error (SE) of triplicate experiments. The activity against Bz-Arg-MCA was considered 100 %.

As shown in Fig. 3, the protease from *D. opposita* “Nagaimo” exhibited high activity toward the synthetic substrate Bz-Arg-MCA and moderate activity toward Pro-Phe-Arg-MCA, Z-Gly-Pro-Arg-MCA, Z-Arg-Arg-MCA, Z-Leu-Arg-MCA, and Z-Phe-Arg-MCA. In addition, the enzyme very weakly hydrolyzed the substrates Boc-Gln-Arg-Arg-MCA, Boc-Val-Leu-Lys-MCA, Z-Val-Val-Arg-MCA, Boc-Glu-Lys-Lys-MCA and Boc-Val-Pro-Arg-MCA. It did not hydrolyze several substrates that are known to be hydrolyzed by other proteases such as cathepsin H or aminopeptidase (Arg-MCA), elastase (Suc-Ala-Ala-Ala-MCA), tripeptidyl peptidase II (Ala-Ala-Phe-MCA) or dipeptidyl peptidase IV (Gly-Pro-MCA). Additionally, the enzyme did also not hydrolyze caspase substrates (Ac-Asp-Asn-Leu-Asp-MCA, Ac-Asp-Glu-Val-Asp-MCA and Ac-Asp-Gln-Thr-Asp-MCA) (data not shown).

These results indicate that the enzyme preferentially cleaves the peptide bond between the P^1^ (Arg or Lys) and P^1’^ positions in the presence of a hydrophobic amino acid such as Leu, Val, or Phe at the P^2^ position, except for Bz-Arg-MCA and Z-Arg-Arg-MCA.

The *K*_*m*_*, V*_max_*, k*_cat_ and *k*_cat_*/K*_m_ values of the purified enzyme for 12 substrates at the optimal pH (pH 8.0) are presented in Table 3. The *K*_m_*, V*_max_*, k*_cat_ and *k*_cat_*/K*_m_ values for Bz-Arg-MCA, which was most preferentially cleaved by the enzyme, were 10.0 μM, 20.83 μmol/mg/min, 28.13 sec^−1^ and 2.81 s^−1^ μM^−1^, respectively. The relative order of *k*_cat_ was as follows; Bz-Arg-MCA > Z-Gly-Pro-Arg-MCA ≥ Z-Arg-Arg-MCA ≥ Pro-Phe-Arg-MCA = Boc-Gln-Arg-Arg-MCA. The relative order of *k*_cat_/*K*_m_ was as follows: Bz-Arg-MCA > Z-Arg-Arg-MCA > Pro-Phe-Arg-MCA > Z-Gly-Pro-Arg-MCA. These results indicated that Bz-Arg-MCA was the optimal substrate among the 12 substrates tested, which was in agreement with the results shown in Fig. 3.

**Table 3:** Kinetic parameters of protease from Nagaimo for different substrates.

Substrate	Km	Vmax	kcat	kcat/Km
	(μM)	(μmol/mg/min)	(sec-1)	(sec-1.μM-1)
Bz-Arg-MCA	10.0	20.83	28.13.	2.81
Pro-Phe-Arg-MCA	10.0	16.67	22.51	2.25
Z-Gly-Pro-Arg-MCA	11.8	17.86	24.12	2.04
Z-Arg-Arg-MCA	10.0	17.27	23.33	2.33
Z-Leu-Arg-MCA	12.5	15.63	21.11	1.67
Z-Phe-Arg-MCA	11.0	11.76	15.88	1.44
Boc-Val-Pro-Arg-MCA	12.5	12.60	17.02	1.36
Boc-Val-Leu-Lys-MCA	10.0	10.00	13.51	1.35
Boc-Gln-Arg-Arg-MCA	15.0	16.67	22.51	1.50
Z-Val-Val-Arg-MCA	10.0	12.50	16.88	1.69
Boc-Glu-Lys-Lys-MCA	5.0	3.33	4.50	0.90
Arg-MCA	17.9	0.18	0.24	0.01

#### 3.2.3 Effect of pH and temperature on protease activity and stability

**Fig. 4:**
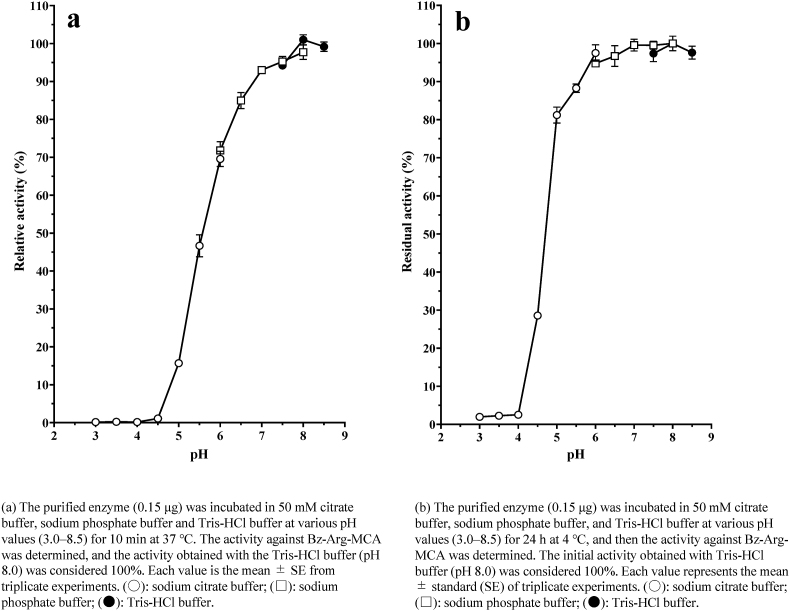

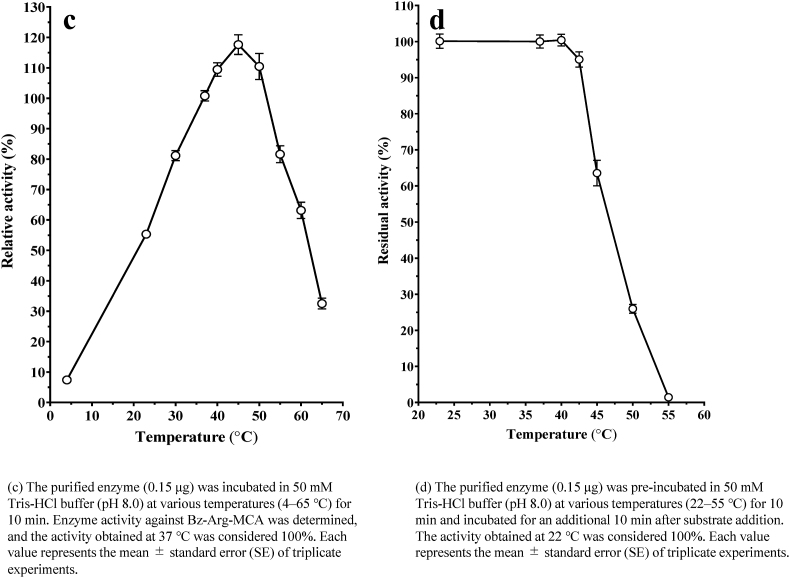
Effect of pH and temperature on the activity and stability of purified protease from *Dioscorea opposita* “Nagaimo”. (a) The purified enzyme (0.15 μg) was incubated in 50 mM citrate buffer, sodium phosphate buffer and Tris-HCl buffer at various pH values (3.0–8.5) for 10 min at 37 °C. The activity against Bz-Arg-MCA was determined, and the activity obtained with the Tris-HCl buffer (pH 8.0) was considered 100 %. Each value is the mean ± SE from triplicate experiments. (○): sodium citrate buffer; (□): sodium phosphate buffer; (●): Tris-HCl buffer. **(b)** The purified enzyme (0.15 μg) was incubated in 50 mM citrate buffer, sodium phosphate buffer, and Tris-HCl buffer at various pH values (3.0–8.5) for 24 h at 4 °C, and then the activity against Bz-Arg-MCA was determined. The initial activity obtained with Tris-HCl buffer (pH 8.0) was considered 100 %. Each value represents the mean ± standard (SE) of triplicate experiments. (○): sodium citrate buffer; (□): sodium phosphate buffer; (●): Tris-HCl buffer. **(c)** The purified enzyme (0.15 μg) was incubated in 50 mM Tris-HCl buffer (pH 8.0) at various temperatures (4–65 °C) for 10 min. Enzyme activity against Bz-Arg-MCA was determined, and the activity obtained at 37 °C was considered 100 %. Each value represents the mean ± standard error (SE) of triplicate experiments. **(d)** The purified enzyme (0.15 μg) was pre-incubated in 50 mM Tris-HCl buffer (pH 8.0) at various temperatures (22–55 °C) for 10 min and incubated for an additional 10 min after substrate addition. The activity obtained at 22 °C was considered 100 %. Each value represents the mean ± standard error (SE) of triplicate experiments.

The activity of the purified protease was determined in a pH range of 3.0–8.5 using sodium citrate (pH 3–6), sodium phosphate (pH 6–8) and Tris-HCl (pH 7.5–8.5) buffers. The protease exhibited maximum activity on Bz-Arg-MCA at an optimal at pH of 7.5–8.5 (Fig. 4a).

The enzyme was incubated at a pH of 3.0–8.5 (in the above mentioned buffers) for 24 h at 4 °C to examine the effect of pH on protease stability, and its residual activity was determined. The enzyme was stable over a wide pH range from 5.5 to 8.5 (Fig. 4b).

The optimal temperature for the hydrolysis of Bz-Arg-MCA by the protease in Tris-HCl buffer (pH 8.0) was approximately 40–45 °C (Fig. 4c).

The enzyme activity was thermostable up to 0–42.5 °C for 10 min (Fig. 4d). In addition, the enzyme was stable on storage in 20 mM Tris-HCl buffer (pH 8.0), the enzyme was stable for at least 7 days at 4 °C and 2 months at −30 °C.

#### 3.2.4 Inhibition of protease activity

**Fig. 5:**
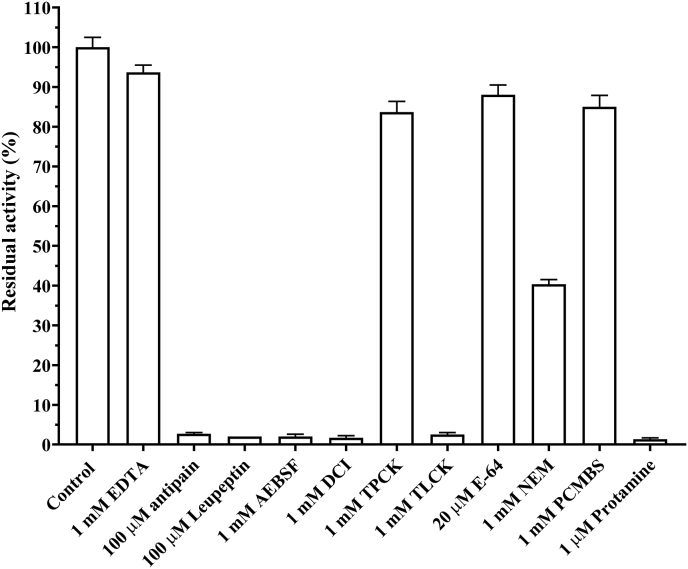
Inhibition of the purified protease from *Dioscorea. opposita* “Nagaimo” by several protease inhibitors. The purified enzyme (0.15 μg) was pre-incubated with each inhibitor for 10 min at 23 °C in 50 mM Tris-HCl buffer (pH 8.0), and the assays began with the addition of 100 μM Bz-Arg-MCA. The reaction mixtures were incubated for an additional 10 min at 37 °C. Each value represents the mean ± standard error (SE) of triplicate experiments.

The ability of various known protease inhibitors to inhibit Nagaimo protease was examined (Fig. 5). Protease activity was strongly inhibited by antipain, leupeptin, AEBSF, TLCK, protamine [27] and DCI [[28], [29], [30]], and moderately inhibited by N-ethylmaleimide (NEM). Enzyme activity was not inhibited by ethylenediaminetetraacetic acid (EDTA), phenylmethylsulfonyl fluoride (PMSF), TPCK, or E−64.

The inhibition patterns of AEBSF, DCI, and TLCK, suggest that this enzyme may belongs to the trypsin-like of serine protease family.

The half-maximal inhibitory concentration (IC_50_) of the above mentioned inhibitors toward the serine protease purified from Nagaimo was determined by assaying the enzyme activity over a range of inhibitor concentrations at a fixed substrate concentration of 100 μM (Table 4).

**Table 4:** IC_50_ values of various inhibitors for protease from Nagaimo.

Inhibitor	IC_50_ (μM)
Leupeptin	0.12
Antipain	0.21
AEBSF	0.70
DCI	30.0
TLCK	0.07
TPCK	no-inhibition
NEM	880.0
E−64	no-inhibition
Protamine	20.5
PCMBS	no-inhibition
EDTA	no-inhibition

The relative order of IC_50_ values for the synthetic inhibitors was TLCK > leupeptin > antipain > AEBSF > protamine > DCI > NEM. The protamine-mediated inhibition of protease activity is theorized to be caused by the presence of polyarginine [27] units in its structure, which act as substrate analogs. In contrast, NEM inhibition causes the binding of a cysteine residue in the protein molecule.

Morty et al. [31] reported that OPB originating from *Trypanosoma brucei* was inhibited by thiol-blocking reagents such as *p*-chloromercuribenzoic acid, NEM, and indole-3-acetic acid (IAA), and its activity was improved by the addition of reducing reagents such as dithiothreitol, glutathione and cysteine. In *Trypanosoma brucei* OPB [31], C^256^ residue in the β-propeller was suggested to be the reactive residue (S1-site or P1 substrate-binding site) that mediates the inhibition of enzymatic activity by NEM and IAA.

Three Cys residues (C^145^, C^272^ and C^356^) in the β-propeller of Nagaimo OPB (Fig. 10) are present, Cys^272^ residue may considered as S1-sites and further study is needed.

#### 3.2.5 Effect of divalent cations on protease activity

**Fig. 6:**
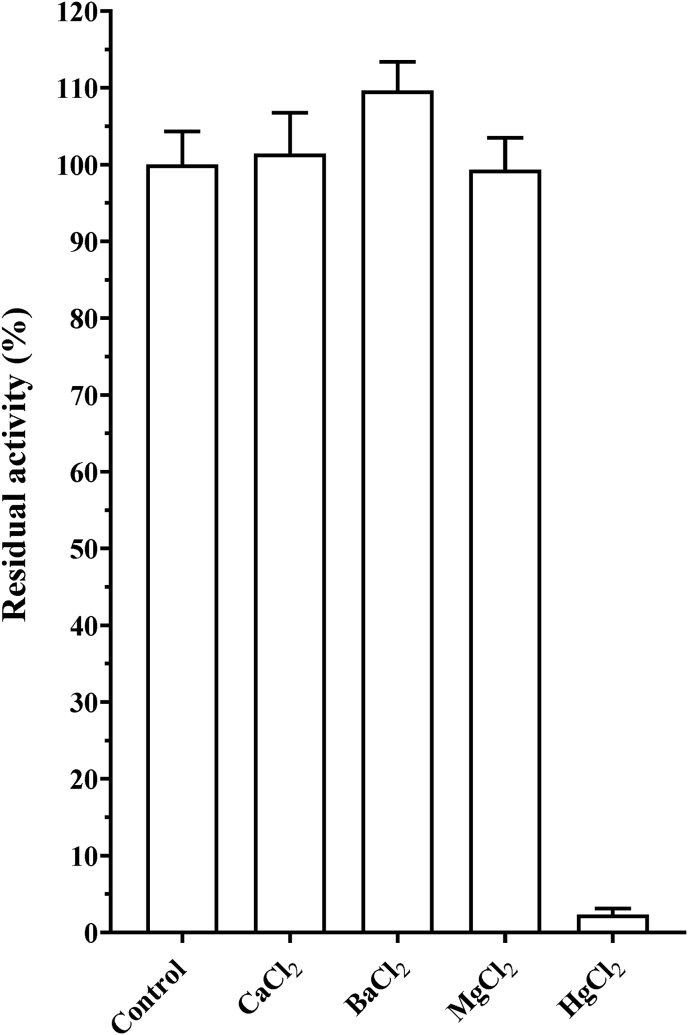
Effect of divalent cations on activity of protease from *Dioscorea oppo*sita “Nagaimo”. Protease assays were performed at 37 °C for 10 min in 50 mM Tris-HCl buffer (pH 8.0) in the presence of 0.15 μg enzyme, 100 μM Bz-Arg-MCA, and 1 mM solution of a divalent cation. Each value represents the mean ± standard error (SE) of triplicate experiments.

**Fig. 7:**
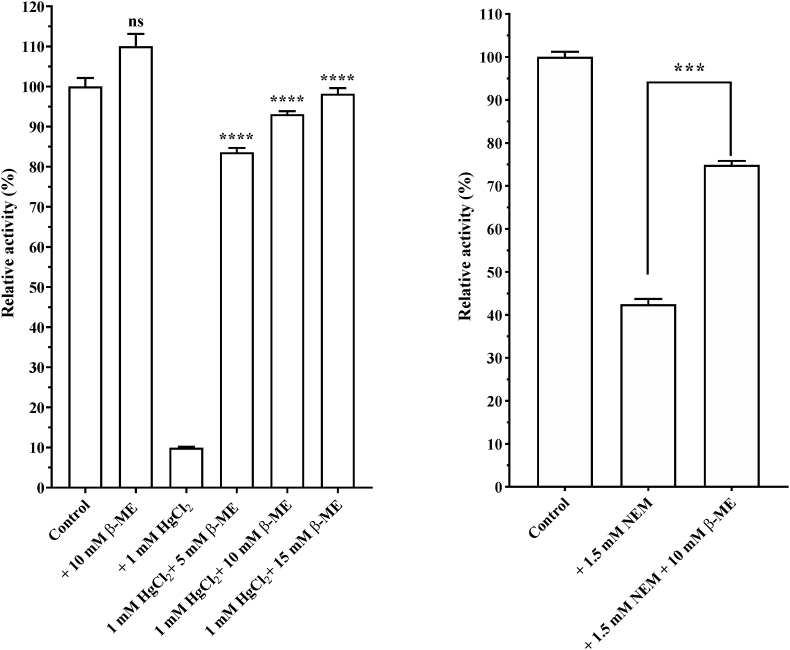
Recovery of protease activity suppressed by HgCl_2_ (left) and NEM (right) Data are expressed as mean residual activity ± SEM (n = 3). Asterisks indicate statistically significant differences between samples with and without β-ME (ns, not significant; ∗∗∗, *p* < 0.001; ∗∗∗∗, *p* < 0.0001).

Among the divalent cations tested, including CaCl_2_, BaCl_2_, MgCl_2_, and HgCl_2_, only HgCl_2_ strongly inhibited Nagaimo protease activity (Fig. 6). Protease activity inhibited by HgCl_2_ was significantly restored by the addition of →-ME in a concentration-dependent manner (*p* < 0.0001) (Fig. 7a). Furthermore, protease activity inhibited by NEM was significantly restored by the addition of β-ME (*p* < 0.001) (Fig. 7b).

HgCl_2_ binds to cysteine residues in proteins and modulates enzyme activity. The enzymatic activity of Nagaimo protease inhibited by NEM and HgCl_2_ recovered by the addition of β-ME, indicating that the mercury- and NEM-binding regions on the enzyme are crucial for the regulation of enzyme activity.

#### 3.2.6 Analysis of the proteolytic effects of purified protease on α-, β-, and γ-caseins

**Fig. 8:**
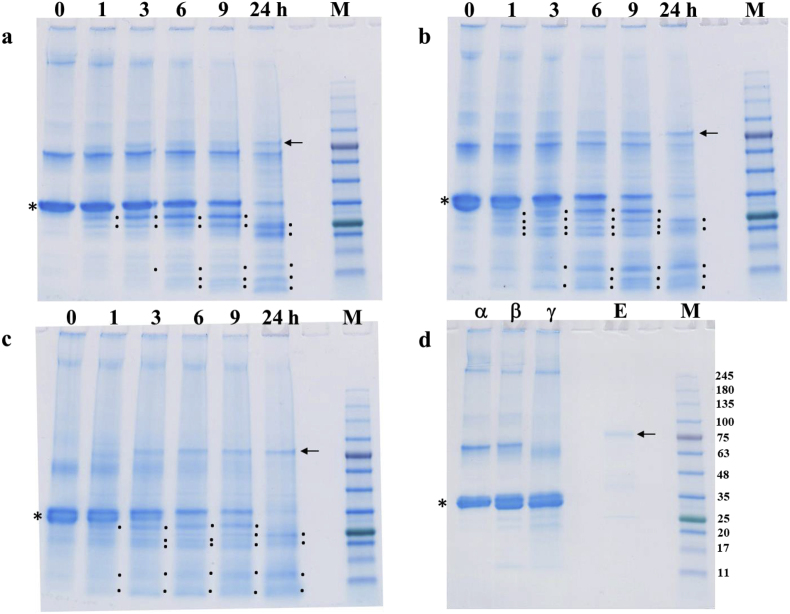
Analysis of the proteolytic activity of purified oligopeptidase B on α-, β- and γ-caseins at an E/S ratio of 1:10 (w/w) from 0 to 24 h. (a), (b) and (c) represent the proteolytic activity of oligopeptidase B on α-, β- and γ-caseins, respectively; (d) shows the substrate and enzyme controls. Asterisks, arrows, dots, and “M” denote caseins, oligopeptidase B, protein bands newly degraded from caseins and standard protein markers, respectively. Zero indicates untreated α-, β- and γ-caseins as controls.

Fig. 8a, b, and 8c indicate the electrophoretic pattern of α-, β-, and γ-caseins, respectively, after treatment with purified protease [enzyme/substrate ratio (E/S) = 1:10] in 20 mM Tris-HCl buffer (pH 8.0) for 1–24 h at 23 °C.

The results indicated that the protease gradually degraded a moderately large portion of α-, β-, and γ-caseins into several 0.5- and 24-kDa peptides over 24 h. Similarly, 80–90 % of α-, β-, and γ-caseins were degraded by OPB in 24 h.

Caseins (α, β and γ-caseins) have molecular weights of 19,000–25,000 (169–209 aa residues) and are known to form micelles in aqueous solution [32,33]. Before these micelles pass through the β-propeller domain and reach the catalytic site, steric hindrance between the protease and the substrate caseins is assumed to occur, resulting in insufficient protease activity.

#### 3.2.7 Determination of N-terminal amino acid and internal amino acid sequences

The N-terminal of the protease was blocked in an unknown manner. However, 78 internal amino acids of the protease were obtained by LC-MS/MS analysis and were compared to the amino acid sequence (**NP 001322305.1**) of the prolyl oligopeptidase family protein originating from *Arabidopsis thaliana* and prolyl OPB (**XP 044442509**) of the wheat embryo *Tritcum aestivum* (data not shown). The internal amino acids showed high homology to the amino acid sequences of the *A. thaliana* (69/78 = 88.46 %) and the wheat embryo *Tritcum aestivum* (70/78 = 89.74 %) oligopeptidases. Among the internal amino acid sequences determined from the Nagaimo protease, the sequence containing the active site serine was SAGGLLMGAVLNMR, which was almost identical to the corresponding sequence in the wheat embryo and *A. thaliana* oligopeptidases, differing in only one amino acid (data not shown).

Furthermore, the nucleotide sequence was determined, and the amino acid sequence of OPB from *D. opposita* “Nagaimo” was deduced using next-generation sequencing and PCR.

**Fig. 9:**
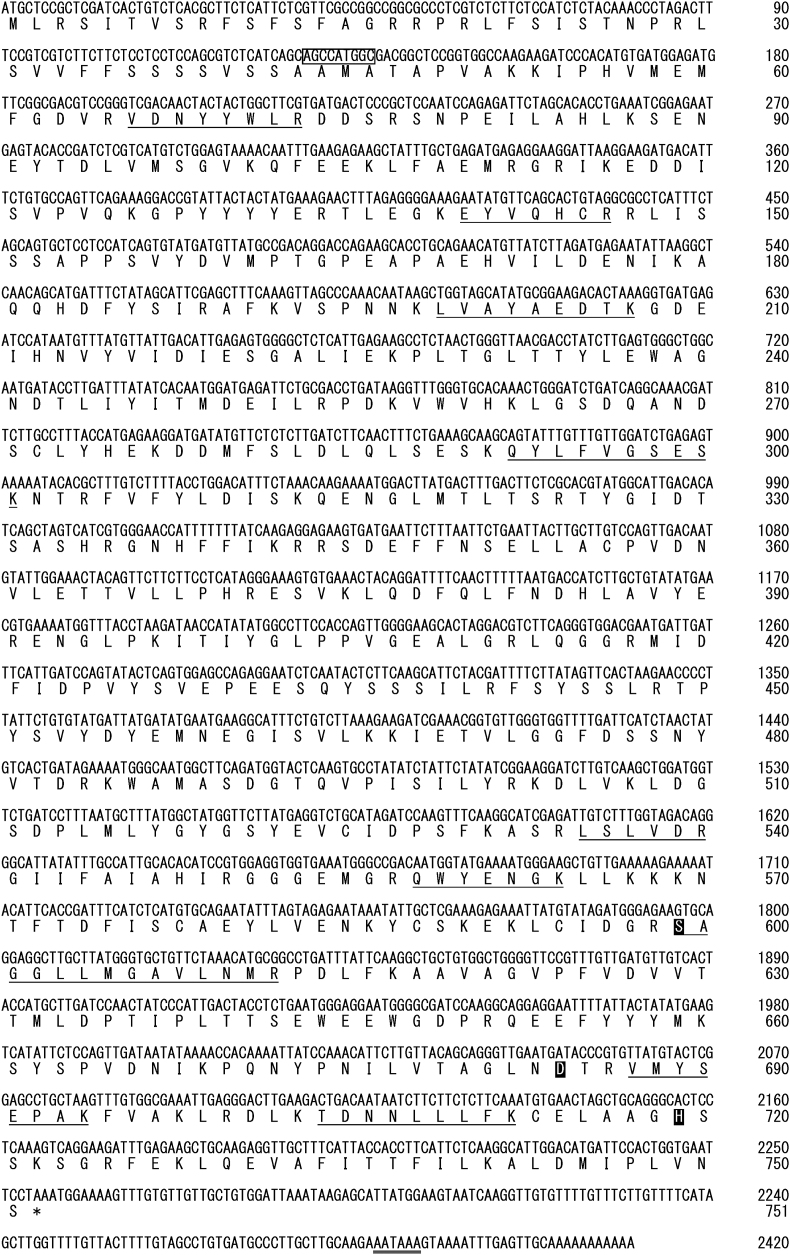
Nucleotide sequence and deduced amino acid sequence of oligopeptidase B of *Dioscorea opposita* ‘Nagaimo’ cDNA. Single-letter notations are used for amino acids. The sequences of derived from trypsin-digested are underlined with single solid lines, and the residues presumably involved in the coordination of a catalytically active site amino acids indicated by Coetzer et al. [34] are enclosed in boxes [S^599^, D^684^ and H^719^]. The boxed nucleotide sequences indicate Kozak [37] or Kozak-like [38] sequences. Polyadenylation sequence of AATAAA is shown by a double underline. An asterisk indicates stop codon.

The nucleotide and deduced amino acid sequences of OPB are shown in Fig. 9. The cDNA (2420 bp) was composed of 2253 bp coding for OPB, a stop codon, 153 bp of a 3′ non-coding sequence, and 11 bp of a poly (A) tail. The polyadenylation or processing sequence of AATAAA was present at 16 bp upstream of the poly (A) tail. The total number of amino acid residues in OPB predicted from the coding region was 751. The amino acid composition of pre-OPB was as follows: Asp_50_, Asn_30_, Thr_36_, Ser_69_, Glu_51_, Gln_18_, Pro_36_, Gly_44_, Ala_40_, Val_53_, Met_21_, Ile_42_, Leu_72_, Tyr_40_, Phe_36_, Lys_45_, His_14_, Arg_39_, Trp_7_, and 1/2-Cys_8_, corresponding to a molecular weight of 85,282. The sequences derived from trypsin digestion are underlined with single solid lines, and the residues presumably involved in the coordination of a catalytically active amino acid site, as indicated by Coetzer et al. [34], are enclosed in boxes [S^599^, D^684^, and H^719^]. The putative amino acid sequence of this enzyme shows the presence of 47 amino acid residues of N-terminal peptide (the true N-terminal amino acids are unknown), a β-propeller domain (419 amino acid residues, P^48^ ∼ K^466^) [35,36] on the N-terminal side, followed by a connecting peptide (61 amino acid residues, K^467^ ∼ D^527^), and then a C-terminal paptidase_S9 domain (217 amino acid residues, P^528^ ∼ D^744^) [34], and a 7-amino acid residue peptide (Fig. 9).

**Fig. 10:**
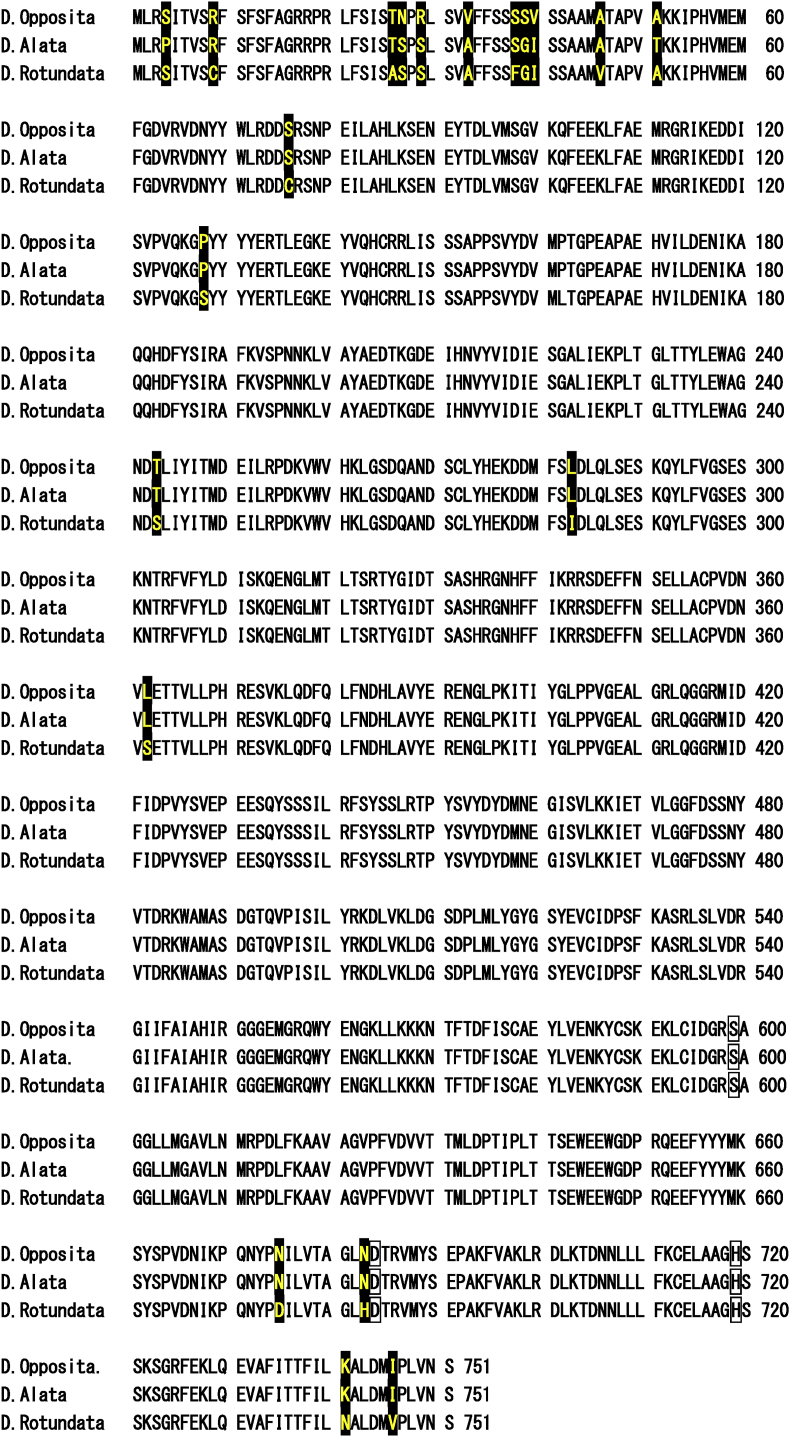
Comparison of the amino acid sequence of prolyl oligopeptidase B originated from *Dioscorea opposita* with that of peptidase S9A prolyl oligopeptidase protein (*D. alata*) and protease 2 (*D. cayenesis* subsp. *rotundata)*. The amino acid sequence of oligopeptidase B of *D. opposita* (GenBank accession no. LC772906) aligns against sequences of *D. alata* (GenBank accession no **KAH7692209.1**) and *D. cayenesis* subsp. *rotundata* (**GeneBank accession no.**XP_039123711.1). Non-preserved amino acid residues among these proteases are shown in filled boxes. Residues presumably involved in the coordination of a catalytically active amino acid site indicated by Coetzer et al. [34] are enclosed in boxes [S^599^, D^684^, and H^719^].

There are four ATG (methionine) sites (M^1^, M^45^, M^58^, and M^60^) on the N-terminal side of this protein, which could be the start codon for translation of this enzyme protein. The Kozak sequence in eukaryotes has been reported as ACCATGG [37] and the Kozak-like sequence in terrestrial plants as AACAATGGC [38], and the sequence near M^45^ is close to both of these sequences. This sequence (AGCCATGGGC) matched the bases in both Kozak sequences. Based on this result, it is highly probable that the ATG at M^45^ is the translation initiation code. Using Met^45^ as the starting point for translation, the molecular weight of OPB was calculated to be 80,350, which gives close agreement with the molecular weight obtained from SDS-PAGE (Fig. 2).

Furthermore, the primary structure of *D. opposita* OPB was highly similar to that of *D. alata* and *D. cayenensis* subsp. *rotundata* (Fig. 10). The levels of amino acid homology were 99.07 % (opposita vs. alata), 97.60 % (*D. opposita* vs. *D. rotundata*). Catalytically active site amino acids [S^599^, D^684^, and H^719^] among these species were well-conserved (Fig. 10).

In particular, comparison of the amino acid sequences of *D. opposita* (Nagaimo) and *D. alata* (Daijo) shows that both sequences from the N-terminal 51st amino acid residue to the C-terminal end (S^751^) are composed of the same amino acid residues. The different amino acids are located in N-terminal side (M^1^ ∼ A^51^, 7/51 = 13.7 %). This data indicates that the enzymes derived from *D. opposita* and *D. alata* have very high genetic relatedness (Fig. 10).

Overall, the substrate specificity, protease inhibitor, and amino acid sequence data indicated that the protease purified from *D. opposit*a “Nagaimo” was an Oligopeptidase B (OPB).

**Fig. 11:**
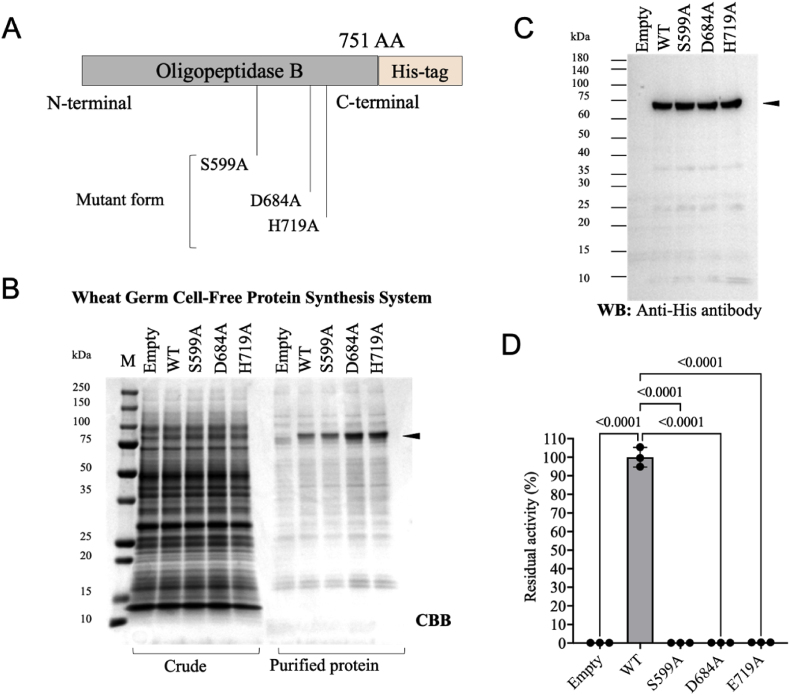
Identification of essential amino acid residues in oligopeptidase B required for hydrolyzing the synthetic substrate Bz-Arg-MCA. **A**. Schematic representation of 6xHis-tagged oligopeptidase B, and the target sequences for site-directed mutagenesis. **B**. SDS-PAGE analysis of crude and purified His-tagged oligopeptidase B (wild-type and mutant forms) purified using Ni-Sepharose beads. All proteins were synthesized using a Wheat Germ Cell-Free Protein Synthesis System. The arrow indicates the predicted molecular weight of oligopeptidase B. M: molecular marker. **C**. Western blot analysis of synthesized proteins using an anti-His-tag antibody. The arrow indicates the predicted molecular weight of oligopeptidase B. **D**. Quantification of proteolytic activity of synthesized proteins against the synthetic substrate Bz-Arg-MCA. Statistical analysis was performed using Dunnett's multiple comparisons test.

To verify the critical roles of S^599^, D^684^, and H^719^ in the catalytic domain of the protease, carboxy-terminal 6xHis-tagged wild-type and alanine-substituted mutant constructs were generated (Fig. 11A). Following confirmation of the mutations by DNA sequencing, the proteins were synthesized using a Wheat Germ Cell-Free Protein Synthesis System [39,40]. The 6xHis-tagged proteins were purified using Ni-Sepharose beads and visualized using SDS-PAGE with Coomassie Brilliant Blue staining (Fig. 11B). To confirm the protein expression at the expected molecular weight, western blotting was performed using an anti-His-tag antibody (Fig. 11C). As expected, oligopeptidase B exhibited protease activity against Bz-Arg-MCA, which was significantly diminished in the alanine mutants (S599A, D684A, and H719A) (Fig. 11D). These results indicated that S^599^, D^684^, and H^719^ in oligopeptidase B were essential for hydrolyzing the synthetic substrate Bz-Arg-MCA.

OPB is expressed in archaea, gram-negative bacteria, eukaryotes, and higher plants [31,34,41], and OPB from Nagaimo may be ancestrally divergent from its bacterial counterparts.

OPB (EC3.4.21.83) belongs to the prolyl oligopeptidase family of serine proteases (clan SC; family S9) (Merops:http://www.merops.ac.uk). The prolyl oligopeptidase family includes many peptidases, including prolyl oligopeptidase (POP; EC 3.4. 21. 26), dipeptidyl peptidase IV (DPPIV, EC 3. 4. 14. 5), OPB (OPB; EC3.4.21.83), and acylaminoacyl peptidase (ACPH; EC3.4.19. 1).

Enzymes belonging to these subfamilies vary in their substrate specificity. POP preferentially hydrolyzes peptide bonds at the C-terminal to proline residues in peptides [42]. In contrast, OPB shows trypsin-like substrate specificity that hydrolyzes peptide bonds on the C-terminal side of basic amino acid residues (arginine and lysine) [43,44].

The enzyme purified in this study revealed to be a trypsin-like protease based on its substrate specificity and the effects of several inhibitors and an OPB based on its amino acid sequence (Table 2, Table 3 and Fig. 9).

As mentioned above, OPB is expressed in bacteria, protists (including *Leishmania* and *Trypanosoma*), eukaryotes, and higher plants; however, its gene has not yet been found in mammals [31]. Protist OPB is a virulence factor that plays a crucial role in the transmission of protozoans to mammals. However, the physiological substrate of protist OPBs remains unknown, and details of its physiological transmission mechanism are not sufficiently understood [31]. The search for and analysis of OPB inhibitors may contribute to the development of new drugs for the treatment of trypanosomiasis.

OPB preferentially cleaves peptides smaller than approximately 30 amino acid residues on the carboxyl side of basic amino acids (Arg or Lys), including adrenocorticotropic hormone, glucagon, dynorphin A, oxidized insulin β-chains, neurotensin, and histone [[43], [44], [45], [46]] Furthermore, Kimec et al. [47] revealed that OPB also degrades smaller peptides with 8–23 amino acid residues. In contrast, OPB from *T. cruzi* has been reported to exhibit no activity against collagen, gelatin, fibrinogen, or fibronectin [48]. The digestion of larger globular proteins depends on the 3D structure of OPB, which comprises an unusual N-terminal seven-bladed →-propeller domain [35,36]. This →-propeller domain has been suggested to block the access of larger globular proteins to the catalytic site of the enzyme [[36], [49], [50]]. However, our results (Fig. 8) showed that 80–90 % of α-, β-, and γ-caseins (Mr 20–25 kDa) were degraded to smaller peptides (0.5–24 kDa) after 24 h. These data indicate that OPB from *D. opposita* “Nagaimo” can degrade proteins with molecular weights similar to those of caseins, albeit at a slower rate.

Furthermore, the protein turnover involving degradation and biogenesis in non-stressed plant cells is highly dynamic, with the half-life of total protein replaced every 4–7 days [50]. In plant cell organelles, OPB degrades the N-terminal signal peptide released by the signal peptidase in the first processing step, and the peptides are produced by trimming the N- and C-terminal, by removing amino acids, cleaving a second sorting signal, and/or cleaving a transmembrane domain, and releasing the soluble portion in the second step.

OPB is also involved in further decomposition of the degradation products produced by the 20S and 26S proteasomes [51]. Tsuji et al. [44] reported that OPB-specific activity was found in germinating embryos and increased 4–5-fold after germination. This suggests that increased protein metabolism depends on the processing or turnover of physiologically crucial peptides rather than on the digestion of storage proteins in the endosperm [44].

However, the significance of OPB in plants remains unknown, and further studies are needed to determine the actual substrates of the enzyme in plant cells and its physio-pathological significance.

## CRediT authorship contribution statement

**Sayaka Miyazaki-Katamura:** Writing – review & editing, Writing – original draft, Resources, Methodology, Investigation, Formal analysis, Conceptualization. **Mami Chosei:** Data curation, Formal analysis. **Sota Tate:** Data curation, Visualization. **Tomohisa Sakaue:** Resources, Methodology, Formal analysis. **Takuya Yamane:** Methodology, Investigation. **Junko Suzuki:** Writing – review & editing, Writing – original draft, Supervision. **Shigeki Higashiyama:** Methodology, Investigation, Formal analysis. **Iwao Ohkubo:** Writing – review & editing, Writing – original draft, Supervision, Methodology, Investigation.

## Declaration of competing interest

The authors declare that they have no known competing financial interests or personal relationships that could have appeared to influence the work reported in this paper.

## Data Availability

Data will be made available on request.
